# Clinical Features, Microbiological Characteristics, and Drug Sensitivity Analysis of Rare Human Spinal Pythiosis Strain

**DOI:** 10.3390/jof10120812

**Published:** 2024-11-22

**Authors:** Mingliang Li, Donglin Zhu, Qiuyue Diao, Xiaoyun Liu, Xiaogang Bi, Jianwen Dong, Jian Sun, Yun Xi, Kouxing Zhang

**Affiliations:** 1 Department of General ICU, The Third Affiliated Hospital of Sun Yat-Sen University, Guangzhou 510630, China; liml3@mail.sysu.edu.cn (M.L.); bixg@mail.sysu.edu.cn (X.B.); 2 Department of Clinical Laboratory, The Third Affiliated Hospital of Sun Yat-Sen University, Guangzhou 510630, China; zhudl3@mail.sysu.edu.cn; 3 National Risk Assessment Laboratory for Antimicrobial Resistance of Animal Original Bacteria, South China Agricultural University, Guangzhou 510642, China; dqy20180412@163.com (Q.D.); jiansun@scau.edu.cn (J.S.); 4 Department of General Medicine, The Third Affiliated Hospital of Sun Yat-Sen University, Guangzhou 510630, China; liuxy59@mail.sysu.edu.cn; 5 Department of Spinal Surgery, The Third Affiliated Hospital of Sun Yat-Sen University, Guangzhou 510630, China; dongjw@mail.sysu.edu.cn

**Keywords:** spinal pythiosis, *Pythium insidiosum*, metagenomic next-generation sequencing, antimicrobial susceptibility, iron chelator

## Abstract

Pythiosis, a rare and formidable infectious disease caused by *Pythium insidiosum*, is characterized by profound uncertainties in achieving definitive diagnoses, suboptimal outcomes, and an exceptionally high mortality rate. Here, we present a rare case of human spinal pythiosis in southern China. With advanced metagenomic sequencing technology, *Pythium insidiosum* was pinpointed as the causative pathogen. We discovered that the inoculation of either tissue fragments or homogenate yielded more successful results and enabled a moderate extension of the culture duration to 5–10 days through an exhaustive comparison of diverse inoculation and culture conditions for general clinical specimens. A pronounced genetic affinity of the isolated strain towards the *Pythium insidiosum* strain MCC 13 was detected after a comprehensive whole-genome sequencing analysis. Antifungal agents exhibited negligible sensitivity towards *Pythium insidiosum* in an antimicrobial susceptibility test. Conversely, antibacterial agents such as oxazolidinones, tetracyclines, macrolides, and amphenicols demonstrated varying degrees of sensitivity, albeit with most of their minimum inhibitory concentrations (MICs) substantially surpassing the safe concentration ranges for effective clinical treatment. Notably, tigecycline stood out as a promising candidate, exhibiting favorable therapeutic effects at moderate concentrations, making it a potential drug of choice for the control of pythiosis. A combined susceptibility test suggested that combinations of tetracyclines with macrolides, oxazolidinones, and amphenicols exhibited synergistic antibacterial effects, with the combination of doxycycline and trimethoprim–sulfamethoxazole (TMP-SMX) in particular playing a pivotal role. To our surprise, the MICs of iron chelators, specifically deferiprone and deferoxamine, against the strain were exceedingly low, which led to the speculation that exogenous iron chelators may have competitively inhibited the iron-chelating enzymes of the strain. The research derived from this single, rare case has certain limitations, but considering that there are currently no reports of invasive infections of deep organs in humans caused by *Pythium insidiosum*, the above findings can offer novel insights into the treatment of invasive pythiosis. Combination therapy based on tetracyclines, especially tigecycline, the use of TMP-SMX, and the adjunctive use of iron chelators, represent promising approaches to tackle the clinical challenges in the treatment of invasive pythiosis. However, further studies, including similar cases of spinal pythiosis and in vivo trials, are still needed to validate them. In addition, while paying attention to the therapeutic potentials of the above plans, we should also closely monitor the risks and side effects that may arise from excessive MICs or the expanded use of related drugs during the treatment process.

## 1. Introduction

Pythiosis, a refractory and extremely rare infectious disease caused by *Pythium insidiosum*, is notorious for its high mortality rate. Its occurrence follows pronounced geographical patterns, primarily affecting tropical, subtropical, and some temperate regions [1,2,3]. The clinical manifestations of pythiosis in humans are diverse and complex. Based on a global analysis of 776 reported cases, corneal pythiosis (574 cases) and vascular pythiosis (169 cases) constitute the overwhelming majority, accounting for a combined proportion of 95.7%, nearly encompassing all known types of cases. Additionally, there are a few instances of skin involvement (22 cases) and disseminated infections (11 cases). Notably, there have been no reported cases of spinal pythiosis to date [4,5,6,7,8,9,10].

For clinicians, the accurate diagnosis and effective management of pythiosis constitute a formidable challenge, possibly due to the non-specific nature of pythiosis’s clinical manifestations. Moreover, the elusive pathogen *Pythium insidiosum*, classified under the Oomycetes taxonomic group [2], mimics fungi morphologically but fundamentally differ in its biological properties. This discrepancy significantly compromises the sensitivity of traditional microbiological diagnostic tools, like smears and cultures, leading to a high potential for misdiagnosis. Furthermore, antifungal therapies frequently demonstrate, more often than not, limited efficacy, resulting in uncertain prognosis and concerning outcomes [11,12,13,14,15]. Our team was at the forefront of reporting the first case of human cutaneous pythiosis in China with an innovative treatment strategy and a favorable therapeutic outcome [5]. Recently, we successfully diagnosed and treated an exceedingly rare instance of human spinal pythiosis. Given that there have been no reports of invasive infections of deep organs caused by *Pythium insidiosum*, this case suggests that the pathogen can cause invasive infections in humans, undoubtedly further expanding the harm of pythiosis. In order to better respond to invasive infections caused by *Pythium insidiosum*, we conducted a comprehensive review of the case and further studied the associated strain, with the objective of unraveling new strategies to address the diagnostic and therapeutic dilemmas posed by pythiosis and providing valuable insights and guidance for clinical practice.

## 2. Materials and Methods

### 2.1. Clinical Data and Isolates

Between July 2022 and June 2023, the patient with spinal pythiosis was investigated in six hospitals in Guangxi and Guangdong Provinces. Patient information and two isolates from positive tissue cultures were collected. This study was approved by the Institutional Research Ethics Committee of the Third Affiliated Hospital of Sun Yat-sen University, and written informed consent was obtained.

### 2.2. Metagenomic Sequencing and Analysis of the Pathogenic Pythium insidiosum Strain

In the course of diagnosis and treatment, diseased tissues such as pus or infected tissue were acquired either through puncture aspiration or surgical resection and subsequently stored in sterile water.

DNA extraction was conducted using the QIAamp^®^ UCP Pathogen Mini Kit (Qiagen, Venlo, The Netherlands), adhering strictly to the manufacturer’s protocol. To eliminate potential contamination from human DNA, a strategic combination of Benzonase (Qiagen) and Tween 20 (Sigma, St. Louis, MO, USA) was employed. Subsequently, the Nextera XT DNA Library Preparation Kit (Illumina, San Diego, CA, USA) was utilized to prepare sequencing libraries from the purified DNA samples. The quality of these libraries was rigorously scrutinized using an Agilent 2100 Bioanalyzer, aided by the Qubit dsDNA HS Assay Kit and High Sensitivity DNA Kit (Agilent, Santa Clara, CA, USA), to guarantee their suitability for subsequent analysis. The pooled libraries were then subjected to sequencing on the Illumina NextSeq 550Dx platform, executing 75 single-end sequencing cycles, yielding approximately 20 million reads per library. This extensive sequencing depth ensured the comprehensive coverage of the microbial diversity present in the samples, facilitating in-depth analysis.

Trimmomatic (Version 0.36) was employed to eliminate low-quality reads, adapter contamination, and duplicate reads, as well as those shorter than 50 bp. Kcomplexity, with its default parameters, was utilized to remove low-complexity reads. Subsequently, the Burrows–Wheeler-Aligner software (Version 0.7.17) was leveraged to map the remaining reads against the human reference genome (hg38), enabling the identification and exclusion of human sequence data. Finally, the purified microbial reads are aligned against reference microbial databases such as NCBI nt and GenBank, facilitating the accurate identification of microbial species.

### 2.3. Morphological Characterization and Growth Curve Analysis of Strains

The harvested lesion tissues were meticulously segmented into minute pieces and directly seeded onto a blood agar plate. Notably, a discernible fraction of the tissue fragments successfully initiated colony formation around their perimeter, exhibiting robust growth. Some colonies were selected for fluorescence staining and lactophenol cotton blue staining.

To further isolate and propagate these strains, the most vigorously growing colonies, identified at the outermost rim of the blood agar plate, were meticulously excised using an agar punch. These selected colonies were then transferred onto Luria–Bertani (LB) agar plates and incubated in a controlled environment at 37 °C.

Daily monitoring of colony growth was conducted, with the diameter of each colony being measured and recorded. These data were utilized to analyze the growth rate of the strains. To ensure the reliability of our findings, each experiment was meticulously replicated three times, and the collected data were subjected to rigorous statistical analysis using SPSS 20.0 software.

### 2.4. Assessment of Strain’s Ability to Induce Sporulation

We selected vigorously growing colonies from the outer edge of an LB agar plate and transferred them onto a 2% Sabouraud dextrose agar plate. We incubated the plate in a 37 °C incubator for 48 h. Colonies from the outer edge were picked and inoculated into centrifuge tubes containing 2% Sabouraud dextrose broth. We placed the tubes in a 37 °C shaker and cultivated them at 180 rpm for 4 h. We removed the broth and replaced it with an equal volume of sterile ultrapure water, continuing the incubation at 37 °C and 180 rpm for 1 h. Subsequently, we discarded the ultrapure water and added an equal volume of sterile induction solution, which primarily comprised a multi-ion solution (K_2_HPO_4_·3H_2_O, KH_2_PO_4_, (NH_4_)_2_HPO_4_, MgCl_2_·6H_2_O, CaCl_2_·2H_2_O) mixed with herbal plant juice. We incubated the mixture at 37 °C and 180 rpm for 16–20 h. Finally, sporulation was observed and recorded under an optical microscope [2,3].

### 2.5. Optimization of Strain Inoculation and Cultivation Methods

Due to the difficulties in culturing *Pythium insidiosum*, we conducted a comparative analysis of six distinct strategies with the aim of identifying the most efficient and suitable inoculation method.

The mycelial plug inoculation group used an agar puncher to precisely excise a vigorously growing colony (a mycelial plug of approximately 6 mm in diameter) from the outer edge of an LB agar plate, and it was directly inoculated onto a blood agar plate. The mycelium suspension inoculation group was formed by emulsifying a mycelial plug with sterilized water and inoculating it onto a blood agar plate. The spore suspension inoculation group was inoculated with motile spores which were induced by sterile induction medium and adjusted to 1–2 × 10^5^ cells/mL. The streaking inoculation group, spreading inoculation group, and punctured inoculation group collected colonies from LB agar plates with inoculation rings, sterile swabs, and inoculation needles, respectively, then inoculated onto blood agar plates.

After inoculation, all plates were closely observed within 24 to 72 h to record and analyze the growth patterns of colonies in each group, aiming to identify the optimal inoculation method for culturing *Pythium insidiosum*.

### 2.6. Assessment of Antimicrobial Susceptibility for Strain

Given the absence of a standardized protocol tailored specifically for *Pythium insidiosum*, we applied various methodologies to evaluate antimicrobial susceptibility testing recommended by the Clinical and Laboratory Standards Institute (CLSI), including the agar diffusion technique, broth microdilution assay, and the radial growth method as previously reported [15,16,17,18,19], each offering unique insights into the strain’s response to antimicrobial agents.

#### 2.6.1. Disk Diffusion Testing

We devised disk diffusion testing based on the agar diffusion technique, using vigorously growing colonies (with mycelial plugs of 6 mm in diameter) as an inoculum. Employing the OXOID disk dispenser (Oxoid Limited, Hants, UK), we evenly distributed test disks containing predetermined quantities of various clinically relevant antimicrobials onto the surface of 90 mm blood agar plates previously inoculated with the colonies. Each disk was positioned 27 mm away from the center of the colony.

After 36–48 h of incubation, the diameter of the inhibition zone around each disk was measured, providing an initial indication of the test organism’s sensitivity to the respective drugs. Each experiment was replicated three times to ensure consistency and reliability.

#### 2.6.2. Broth Microdilution Method

We adapted the broth microdilution method described in the literature [15,16,17,18], utilizing either zoospores or mycelial suspensions as an inoculum. The generated zoospores were resuspended in ultrapure water to a final concentration of 1–2 × 10^4^ cells/mL. For the mycelial suspension, the most peripheral and actively growing colonies from the LB agar plates were taken using an agar puncher, diluted with ultrapure water, vigorously vortexed for 45 s to emulsify them into a uniform suspension, and adjusted to achieve a 85–90% transmittance at a 600 nm wavelength.

Mueller–Hinton broth was prepared in a 96-well microdilution plate, containing various commonly used clinical antimicrobial agents’ concentrations (including a no-drug control). Each well was inoculated with 100 μL of the zoospore or mycelial suspension and incubated at 37 °C for 16–48 h. Subsequently, the minimum inhibitory concentration (MIC) and 50% minimum effective concentration (MIC50) were visually determined. The MIC represents the lowest drug concentration that inhibits 100% growth compared to the no-drug control, whereas the MIC50 indicates the lowest drug concentration causing at least 50% growth inhibition. Each experiment was repeated three times for reproducibility.

#### 2.6.3. Combined Antimicrobial Susceptibility Testing

To further elucidate the effects of drug combinations on the strain in this case, we performed a comprehensive combined antimicrobial susceptibility screening test after determining the individual inhibition zone of the relevant drugs, integrating both clinical data and disk diffusion susceptibility outcomes [15,16].

The aforementioned mycelial suspension was selected as the inoculum for this experiment. Antimicrobial drug disks, representing various antibiotics, were strategically positioned on the surface of a 90 mm blood agar plate inoculated with the mycelial suspension. The positioning was guided by a reference distance of 3–4 mm between the edge of one disk and the edge of the zone of inhibition formed by another disk. Following incubation, the interaction between the two drugs was assessed by observing phenomena such as the expansion or contraction of the zones of inhibition between them. These observations enabled us to categorize the interactions as synergistic, additive, indifferent, or antagonistic.

### 2.7. Strain Whole-Genome Sequencing Analysis

To facilitate comprehensive genomic analysis, actively growing colonies were selected and subjected to a rigorous extraction protocol. Specifically, 500 μL of lysis buffer (comprising Tris, EDTA, NaCl, β-mercaptoethanol and 20% SDS) was administered to the colonies. This mixture was then lysed at 65 °C for 15 min, and this was followed by the addition of 500 μL of DNA extraction solution (comprising phenol, chloroform, and isoamyl alcohol) to eliminate impurities, and the mixture was subsequently centrifuged at 12,000 rpm for 10 min to yield a clarified supernatant. This purification step was repeated twice. Then, double-volume isopropanol was added to the supernatant. The mixture was incubated at −20 °C for 30 min and centrifuged at 12,000 rpm for 10 min to precipitate the genomic DNA. The precipitate was washed twice with 75% ethanol, resulting in the isolation of purified genomic DNA.

Upon quality verification, the DNA samples underwent random fragmentation using a Covaris ultrasonic disruptor (Covaris, Woburn, MA, USA). This fragmented DNA then underwent a series of meticulous steps including end-repair, A-tailing, the ligation of sequencing adapters, size selection, PCR amplification, and purification to create the final DNA library. Utilizing the Agilent 5400 system (AATI), the quality of the library was assessed, and qualified libraries were further processed through bridge PCR on a FlowCell chip (Illumina, San Diego, CA, USA). Employing the Illumina NovaSeq 6000 platform with paired-end sequencing, base calling and Bcl2Fastq conversion yielded raw sequenced reads in the FASTQ format.

To prepare the raw data for downstream analysis, fastp (Version 0.23.1) was employed to preprocess the raw reads, resulting in clean reads suitable for subsequent analyses.

The genomic assembly of the clean reads was achieved using MEGAHIT (Version 1.2.9), with the assembly quality assessed by QUAST (Version 5.2.0). MLST software (Version 2.23.0) was utilized for the sequence typing of the genome, while PlasmidFinder (Version 2.0.1) and Platon (Version 1.6) identified plasmid types. Genome annotation was conducted for each assembly using Prokka (Version 1.14.5), and CGView (Version 2.0.3) served as a comparative tool for genomic visualization.

To gain further insights, OrthoFinder (Version 2.5.5) was applied to identify single-copy genes, and MUSCLE (Version 5.1) was employed for multiple sequence alignment of the protein-coding genes. Conserved sites were identified using Gblocks (Version 0.91b), and subsequently, a phylogenetic tree was constructed using PhyML (Version 3.3) under the maximum likelihood framework.

## 3. Results

### 3.1. Case Presentation

In July 2022, a 48-year-old Chinese male patient with a history of chronic hepatitis B and thalassemia developed a high fever, pus discharge from the left ear, and headaches after experiencing ear water ingress while swimming and navigated through several local medical institutions without any notable improvement. During hospitalization, the patient underwent multiple conventional microbiological examinations, all of which yielded negative results, except for the tuberculosis antibody testing positive. He was administered various antibiotics, including cefuroxime, ceftriaxone, cefoperazone/sulbactam, linezolid, and moxifloxacin, for infection control, but the outcomes were unsatisfactory. Subsequently, he gradually developed neck pain and difficulty in neck movement. Magnetic resonance imaging (MRI) revealed “enlarged cervical lymph nodes and swelling of the soft tissues in the neck”, prompting his transfer to a higher-level hospital in Guangzhou for further treatment.

Upon transferring, his blood test results indicated a significant reduction in hemoglobin levels (71 g/L), while his white blood cell count (6.12 × 10^9^/L) and platelet count (109 × 10^9^/L) were within normal ranges. His coagulation indices remained unremarkable, but his albumin levels were decreased (33.18 g/L). Immunological markers showed a mild enhancement of his immune response (IgG 31.46 g/L, IgA 8.88 g/L, IgM 1.07 g/L, C3 1.40 g/L, C4 0.34 g/L) and inflammatory level (procalcitonin < 0.01 ng/mL, C-reactive protein 13.46 mg/L, interleukin-6 88.67 pg/mL, erythrocyte sedimentation rate 120 mm/h). Notably, his ferritin (FER) levels were significantly high (1642.15 ng/mL). GM and G tests yielded results of 0.07 s/co and 96.84 pg/mL, respectively. Meanwhile, a PPD test and TB-SPOT test showed positive results. MRI scans suggested possible abscess formation in the cervical, thoracic, and adjacent soft tissues. Then, a cervical lymph node biopsy was performed on 19 October 2022, and a cervical paravertebral abscess biopsy was performed on 25 October 2022. However, both pathogen tests were negative, and pathological results revealed the slight infiltration of inflammatory cells with negative acid-fast (AF) staining. Based on the chronic inflammatory lesions and abnormal tuberculosis screening results, clinicians initially considered the possibility of a cervical vertebrae tuberculosis abscess. However, subsequent treatments with linezolid, moxifloxacin, levofloxacin, and imipenem were ineffective, and the patient continued to experience low-grade fever and persistent neck and back pain; thus, he was transferred again.

The follow-up blood tests revealed minimal changes from previous results, with a high serum FER remaining (2459.58 ng/mL). MRI scans indicated infectious spondylitis of the cervical and upper thoracic spine with multiple abscess formations in the intervertebral disks and prevertebral spaces. Then, cervical paravertebral abscess puncture was performed on 8 November 2022 and C3/4-C5/6 prevertebral/intervertebral/intraspinal lesions removal was performed on 16 November 2022. Finally, the lesions were sent for microbiological, pathological, and mNGS testing.

### 3.2. Identification of Pythium insidiosum Through Microbiology, Pathology, and mNGS

During the patient’s first to fourth hospitalizations across multiple local hospitals, repeated microbiological tests of bodily fluids (blood/sputum/urine/stool/pus) consistently yielded negative results, and mNGS was not performed.

During the fifth hospitalization at Hospital I in Guangzhou, cervical lymph node and paravertebral abscess aspirations were conducted on days 108–119 of onset and still revealed the slight infiltration of inflammatory cells with negative pathogen detection and AF staining.

During the sixth hospitalization at Hospital II in Guangzhou, paravertebral cervical abscess aspiration was performed on day 125 of illness (5 November 2022). Tissue culture results were negative, while mNGS detected the presence of *Pythium insidiosum*, with 48 sequences identified. On day 136 (16 November 2022), prevertebral/intervertebral/intraspinal lesions debridement at C3/4-C5/6 was performed, and the tissue culture with bedside inoculation was positive; pathological examination suggested chronic suppurative inflammation with abscess formation, and mNGS confirmed the infection with 1202 *Pythium insidiosum* sequences. However, during a C6/7-T2/3 intervertebral disk lesionectomy on day 177 (27 December 2022), tissue culture was again negative, while histopathological examination revealed chronic active inflammation, with negative results for AF staining, Grocott’s methenamine silver (GMS) staining, and periodic acid–Schiff (PAS) staining.

During the seventh hospitalization at Hospital II in Guangzhou, on day 482 (27 October 2023), C7, T4/5-T7/8, and T11 intervertebral disk and paravertebral lesion debridement was performed. Tissue culture suggested the *Pythium* species, and histopathological examination revealed chronic suppurative inflammation with partial positivity for CD68, Kappa (+), and Lambda (+), but mNGS failed to detect *Pythium insidiosum* sequences.

Finally, during the eighth hospitalization at Hospital II in Guangzhou, on day 573 (26 January 2024), T9/10-T10/11 intervertebral disk and spinal canal infection lesion debridement was performed. Tissue culture again indicated *Pythium* species, and histopathological examination showed a vertebral abscess with negative AF staining, GMS staining, and PAS staining, and mNGS confirmed the presence of *Pythium insidiosum*, identifying 624 sequences (Table 1).

### 3.3. Antimicrobial Treatment and Outcome

**Figure 1:**
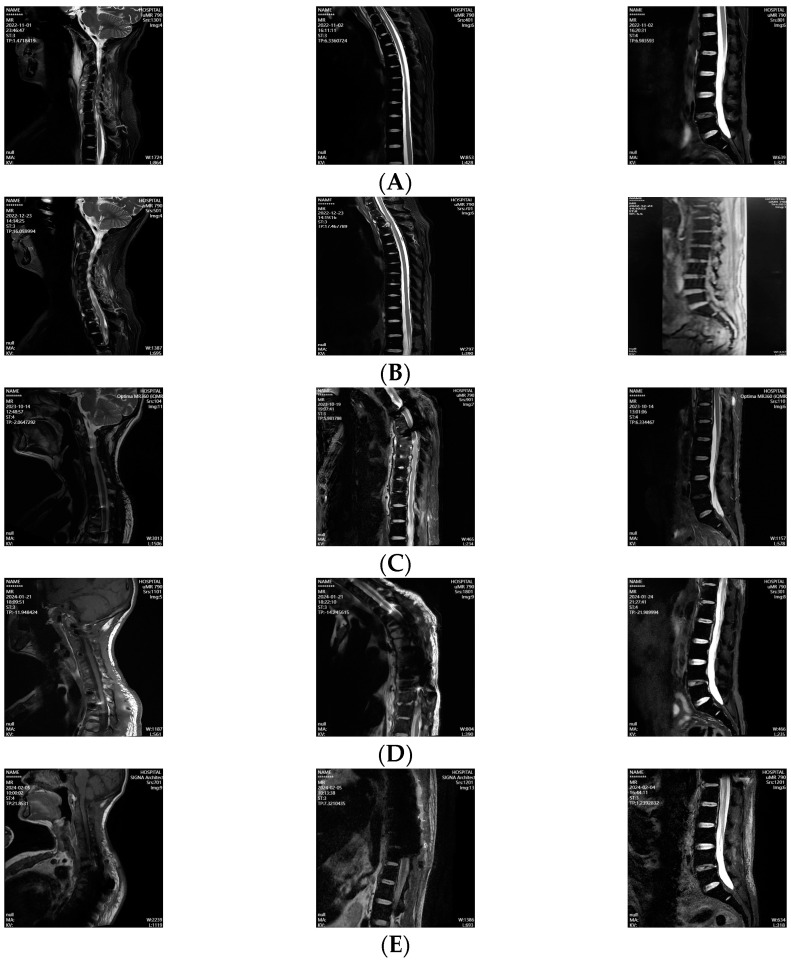
MRI scan of cervical, thoracic, spine, and lumbar spine. (**A**) Pre-first surgery (November 2022). (**B**) Pre-second surgery (December 2022). (**C**) Pre-third surgery (October 2023). (**D**) Pre-fourth surgery (January 2024). (**E**) Follow-up imaging (February 2024).

Based on the results of an MRI scan (Figure 1A) and microbiological, pathological, and mNGS testing, a multidisciplinary consultation considered it a rare human spinal pythiosis. It was decided that intravenous azithromycin (0.5 g QD) and oral doxycycline (100 mg Q12H) should be administered for treatment. One week later, a debridement of the C3/4-C5/6 prevertebral, intervertebral, and intraspinal lesions was performed. After 3 weeks, the patient experienced hearing loss, and an adverse drug reaction could not be excluded. Based on previous treatment protocols for skin and subcutaneous pythiosis [5], the azithromycin was discontinued and replaced with oral TMP-SMX (containing 80 mg of trimethoprim and 400 mg of sulfamethoxazole per tablet) administered four times daily, with two tablets each time, combined with the continued use of doxycycline.

However, we found that the ESR rose again to 120 mm/h. A follow-up MRI scan in December 2022 indicated that the lesion had extended to the bilateral brachial plexus and the epidural space at the C6-T1, with infectious changes in the T1-6 vertebral bodies (Figure 1B). Then, a C6/7-T2/3 intervertebral disk lesionectomy was performed.

Due to significant abdominal distension, the patient discontinued medication voluntarily in July 2023. Three months later, a recurrent low-grade fever and pain in the chest and back appeared. A repeat MRI scan revealed infectious lesions in the T1-L1 vertebral bodies, involving the paravertebral tissues at the T10-T11 and the epidural space at the T6–8 levels (Figure 1C). The patient was given TMP-SMX and doxycycline again, followed by a debridement procedure on the C7, T4/5-T7/8, and T11 intervertebral disks and adjacent paravertebral lesions.

**Figure 2:**
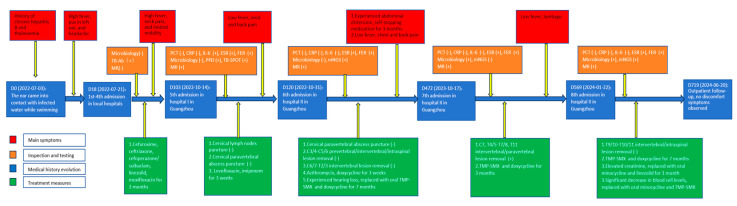
Flowchart of the diagnosis and treatment.

During the follow-up, our patient experienced a recurrent low-grade fever and lumbar pain, and an MRI scan showed infectious lesions in the thoracic vertebral bodies and epidural space of the spinal canal (Figure 1D). In January 2024, a debridement procedure was performed to remove the infectious foci from the T9/10-T10/11 intervertebral disks and spinal canal. Meanwhile, a combination of doxycycline and TMP-SMX was prescribed for anti-infection therapy. However, the subsequent increase in creatinine promoted the discontinuation of doxycycline and TMP-SMX. Instead, oral minocycline (100 mg Q12H) and linezolid (600 mg Q12H) were prescribed. Unfortunately, the blood cell counts were significantly reduced (WBC 2.81 × 10^9^/L, HGB 56 g/L, PLT 94 × 10^9^/L), with an FER at 951.18 ng/mL, leading to the discontinuation of linezolid and continuation of oral minocycline with TMP-SMX. At present, there have been no discomfort symptoms observed during the latest follow-up (Figure 2), and an MRI scan indicated the absorption of cervical lesions, the swelling of paravertebral soft tissue in the thoracic region, and a subcutaneous fascial edema in the lumbosacral region (Figure 1E).

### 3.4. Microbiological and Morphological Characteristics of the Strain

**Figure 3:**
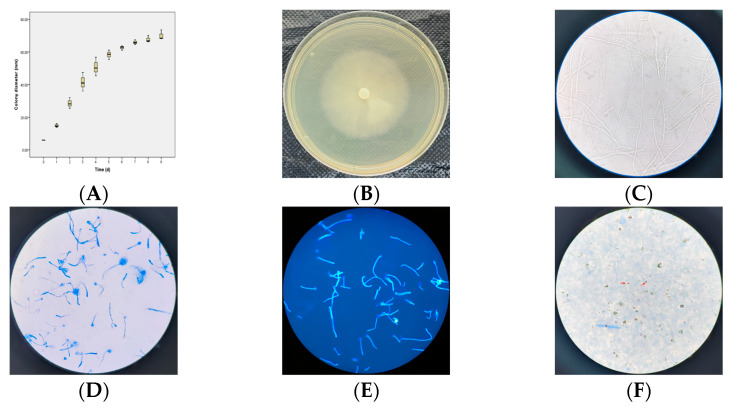
Microbiological morphology of *Pythium insidiosum*. (**A**) Growth curve of the *Pythium insidiosum* inoculated onto LB agar plates. (**B**) Morphology of the strain after 4 days of incubation at 37 °C on a 90 mm LB agar plate. (**C**) Morphology of the mycelium observed under an optical microscope (400×). (**D**) A lactophenol cotton blue staining image of the mycelium (400×). (**E**) A fluorescence staining image of the mycelium (400×). (**F**) Induced zoospore formation in the mycelium observed under an optical microscope (400×). Arrow indicates that the motile spores transformed into spherical shapes, with the loss of flagella.

Upon inoculation onto the LB agar plates and incubation at 37 °C, the strain exhibited an expansive growth pattern on the agar surface after 48 h, with a notable decline in the growth rate commencing on the fifth day post-inoculation. By the tenth day, the colony had fully covered a 90 mm Petri dish (Figure 3A). The colonies appeared white and flat and featured radial grooves, with the mycelium growing in a depressed manner and displaying sparse aerial hyphae (Figure 3B).

Microscopic examination under an optical microscope revealed slender mycelial hyphae, ranging in diameter from 4 to 10 µm. These hyphae showcased vertical branching, sparse septation, and rounded tips, offering a clear picture of their morphological intricacies (Figure 3C). Furthermore, both lactophenol cotton blue staining and fluorescent staining techniques effectively highlighted the sparse septation of the hyphae and the presence of sporangia, enhancing our understanding of their structural features (Figure 3D,E).

Upon stimulation with specific induction solution as previously mentioned, which was a mixture of multi-ion solution and herbal plant juice, the mycelial sporangia underwent a fascinating transformation, releasing motile spores. These spores, adorned with two asymmetrical flagella emerging from lateral grooves, moved in a spiral fashion, displaying their dynamic nature. As the flagella were lost, the motile spores transformed into spherical shapes, further demonstrating their adaptability and versatility (Figure 3F, indicated by the red arrow). These observations collectively contribute to a comprehensive understanding of the microbiological and morphological characteristics of the isolate under investigation.

### 3.5. Comparison of Cultivation Methods

**Figure 4:**
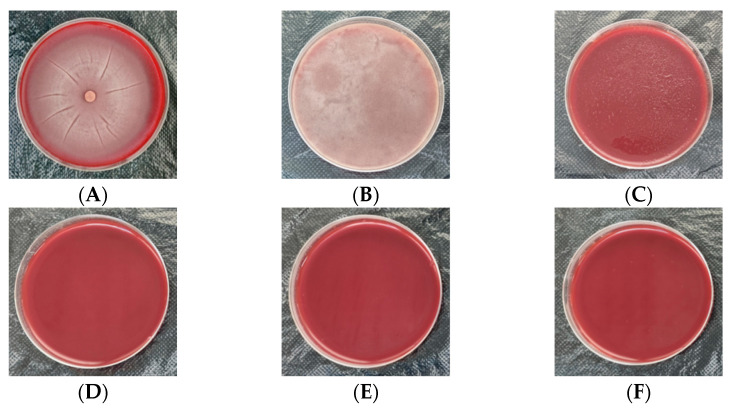
Cultivation of *Pythium insidiosum* (48–72 h). (**A**) Visible robust colony growth after mycelial plug inoculation. (**B**) Apparent colony growth after mycelial suspension inoculation. (**C**) No colony growth observed after spore suspension inoculation. (**D**) No colony growth observed after streak plate inoculation. (**E**) No colony growth observed after spread plate inoculation. (**F**) No colony growth observed after puncture inoculation.

We observed that both the mycelial plug inoculation (Figure 4A) and mycelial suspension inoculation (Figure 4B) resulted in robust colony growth on the blood agar plates within 24 h. By 48–72 h, the 90 mm plates were fully covered, with the mycelial suspension inoculation exhibiting a more optimal growth. In contrast, no colony growth was observed within 48–72 h in groups applying other techniques, including spore suspension inoculation (Figure 4C), streak plate inoculation (Figure 4D), spread plate inoculation (Figure 4E), and puncture inoculation (Figure 4F).

### 3.6. Assessment of Strain Antimicrobial Susceptibility Testing (Table 2)

#### 3.6.1. Test Results of Disk Diffusion

Drug-impregnated disks were dispensed onto the surface of 90 mm blood agar plates. Following incubation at 37 °C for 16–48 h, inhibition zones around the disks were observed for various antimicrobials. The diameter of these zones, in descending order, were as follows: linezolid (19.95 mm), minocycline (19.75 mm), tigecycline (15.47 mm), doxycycline (11.76 mm), azithromycin (10.10 mm), erythromycin (7.00 mm), tetracycline (7.00 mm), and chloramphenicol (7.00 mm). These belonged to the classes of oxazolidinones, tetracyclines, macrolides, and phenicols. Notably, the oxazolidinones and tetracyclines exhibited significantly larger inhibition zones compared to others. Drugs without discernible inhibition zones primarily comprised β-lactams, lincosamides, quinolones, aminoglycosides, polypeptides, rifamycins, sulfonamides, nitrofurans, and streptogramins.

**Table 2:** Antimicrobial susceptibility testing results.

Classfication	Drug Name	Inhibition Zone (mm)	MIC (μg/mL)	MIC50 (μg/mL)
**1. Antifungal Drugs**				
1.1 Azoles	Itraconazole	NA	>4	
	Voriconazole	NA	>8	
	Fluconazol	NA	>128	
	Posaconazole	NA	>32	
1.2 Polyenes	Amphotericin B	NA	>16	
1.3 Echinocandins	Mikafungin	NA	>32	
1.4 Allylamines	Naphthotifen	NA	>4	
1.5 Other	5-Fluorocytosine	NA	>16	
**2. Antibacterial Drugs**				
2.1 β-Lactams				
2.1.1 Penicillins	Penicillin P	6.00	>256	
	Ampicillin	6.00	>16	
	Piperacillin	6.00	>64	
2.1.2 Cephalosporins	Ceftazolin	6.00	>16	
	Cefotaxiphene	6.00	>8	
	Cefuroxime	6.00	>16	
	Cefotaxime	6.00	>32	
	Ceftazidime	6.00	>16	
	Cefatriaxone	6.00	>32	
	Cefepime	6.00	>16	
2.1.3 Cephalomycins	Cefoxitin	6.00	>4	4
2.1.4 Monocyclic β-Lactam	Amtrazumab	6.00	>16	
2.1.5 Carbapenems	Meropenem	6.00	>8	
	Imipenem	6.00	>8	
	Etapenem	6.00	>1	
2.1.6 β-Lactamase Inhibitors	Amoxicillin/Clavulanic Acid	6.00	>4/2	
	Cefotaxime/Clavulanic Acid	6.00	>4/4	
	Ceftazidime/Clavulanic Acid	6.00	>2/4	
	Piperacillin/Tazobactam	6.00	>64/8	
	Ampicillin/Sulbactam	6.00	>16/8	
	Cefoperazone/Sulbactam	6.00	NA	
	Ceftazidime/Avibactam	6.00	NA	
2.2 Macrolides	Erythromycin	7.00	8	0.5
	Azithromycin	10.10	8	0.5
2.3 Tetracyclines	Tetracycline	7.00	>8	4
	Doxycycline	11.76	4	
	Minocycline	19.75	NA	
	Tigecycline	15.47	≤2	
2.4 Lincosamides	Clindamycin	6.00	>4	
2.5 Quinolones	Ciprofloxacin	6.00	>2	
	Levofloxacin	6.00	>4	
	Moxifloxacin	6.00	>4	
2.6 Aminoglycosides	Gentamicin	6.00	>32	
	Tobramycin	6.00	>4	
	Amikacin	6.00	>32	
	Kanamycin	6.00	>4	
2.7 Oxazolidinones	Linezolid	19.95	>4	2
2.8 Polypeptides	Vancomycin	6.00	>16	
	Teicoplanin	6.00	NA	
	Colistin	6.00	>4	
	Daptomycin	NA	>4	
2.9 Rifamycins	Rifampicin	6.00	>2	
2.10 Sulfonamides	TMP-SMX	6.00	>2/38	
2.11 Phenicols	Chloramphenicol	7.00	16	
2.12 Nitrofurans	Nitrofurantoin	NA	>64	
2.13 Streptogramins	Quinupristin/Dalfopristin	NA	>2	0.5
**3. Other Drugs**	Deferoxamine	NA	0.02	
	Deferiprone	NA	≤0.01	
**4. Combination Drugs**	Doxycycline + Erythromycin	synergistic effect	NA	
	Doxycycline + Azithromycin	synergistic effect	NA	
	Doxycycline + Linezolid	synergistic effect	NA	
	Doxycycline + Chloramphenicol	synergistic effect	NA	
	Doxycycline + TMP-SMX	synergistic effect	NA	
	Minocycline + Erythromycin	synergistic effect	NA	
	Minocycline + Azithromycin	synergistic effect	NA	
	Minocycline + Linezolid	synergistic effect	NA	
	Minocycline + Chloramphenicol	synergistic effect	NA	
	Minocycline + TMP-SMX	indifferent effect	NA	
	Tigecycline + Erythromycin	synergistic effect	NA	
	Tigecycline + Azithromycin	synergistic effect	NA	
	Tigecycline + Linezolid	synergistic effect	NA	
	Tigecycline + Chloramphenicol	synergistic effect	NA	
	Tigecycline + TMP-SMX	indifferent effect	NA	

NA: data not available.

#### 3.6.2. Test Results of Broth Microdilution 

Within the safe concentration ranges of effective clinical treatment, among the major antifungal agents, only the polyene amphotericin B (16 μg/mL) demonstrated mild inhibitory activity, whereas the azoles, echinocandins, allylamines, and 5-fluorocytosine showed no inhibition. Among the antibacterial agents, the tigecycline, doxycycline, erythromycin, azithromycin, and chloramphenicol had defined MIC values. The quinupristin-dalfopristin, linezolid, tetracycline, and cefoxitin exhibited moderate inhibitory effects with established MIC50 values. Notably, the clinically used iron chelators deferoxamine and deferiprone demonstrated exceptionally low MIC values, indicating potent inhibition.

#### 3.6.3. Test Results of Drug Combinations 

Informed by clinical insights and individual drug susceptibility results, a combined antimicrobial susceptibility test was performed, focusing on tetracycline-based dual drug combinations. The following combinations were found to exhibit synergistic effects: doxycycline + erythromycin, doxycycline + azithromycin, doxycycline + linezolid, doxycycline + chloramphenicol, doxycycline + TMP-SMX, minocycline + erythromycin, minocycline + azithromycin, minocycline + linezolid, minocycline + chloramphenicol, tigecycline + erythromycin, tigecycline + azithromycin, tigecycline + linezolid, and tigecycline + chloramphenicol. In contrast, minocycline + TMP-SMX and tigecycline + TMP-SMX displayed indifferent effects.

### 3.7. Whole-Genome Sequencing Analysis of Pythium insidiosum

**Figure 5:**
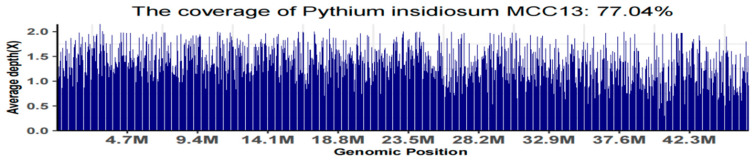
The coverage map of the *Pythium insidiosum* strain MCC 13.

**Figure 6:**
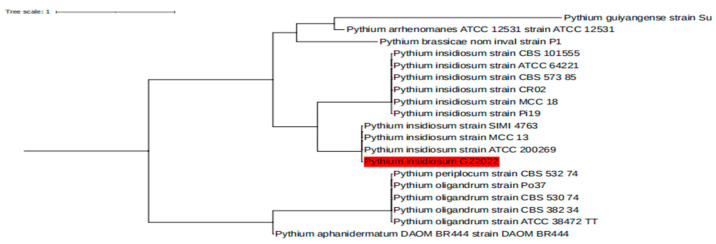
Phylogenetic trees show the phylogenetic position of the sample in this study within the evolutionary radiation of the genus *Pythium*. The highlighted red part represents the strain isolated from tissue biopsy, which showed stronger similarity with the *Pythium insidiosum* strain MCC 13 through blastn sequence alignment analysis.

Comprehensive whole-genome sequencing was conducted on the strain isolated from tissue biopsy and designated as *Pythium insidiosum* GZ2022. The quality assessment of the sequencing data yielded the following key findings: The total number of raw reads amounted to 10,828,570, with 10,513,888 clean reads. The total raw base was approximately 1.62 gigabases (Gb), while the clean base totaled approximately 1.58 Gb. In terms of data quality, the Q20 ratio reached an impressive 98.17%, and the Q30 ratio also surpassed 95.09%, and the GC content was 54.35%. Through blastn sequence alignment analysis, we successfully identified the whole-genome sequence of this strain as comprising 29,424 contigs, with its genomic sequence features showing a stronger resemblance to the *Pythium insidiosum* strain MCC 13 (NCBI link: https://ftp.ncbi.nlm.nih.gov/genomes/all/GCA/001/950/795/GCA_001950795.1_Pythium_insidiosum_MCC13_0.1 (accessed on 6 June 2024)). To provide a more intuitive visualization of the sequencing data coverage, a coverage map was generated (as shown in Figure 5). Furthermore, an evolutionary tree analysis based on single nucleotide polymorphisms (SNPs) was performed using the kSNP4 tool, with the results presented in Figure 6. In-depth analysis revealed that over half of the strain’s genome exhibits a high degree of similarity to the reference genome of the *Pythium insidiosum* strain MCC 13_005477, with a similarity exceeding 97.5%. 

## 4. Discussion

Pythiosis is a highly fatal and refractory infectious disease, with *Pythium insidiosum*, an aquatic Oomycete-resembling fungi, being its sole pathogen in mammals [1,2,3]. Since the first human case was reported in 1985 from Siriraj Hospital, Mahidol University, Thailand [20], subsequent reports of human pythiosis both domestically and internationally have remained scarce. This could be attributed to its non-specific clinical manifestations, difficulties in pathogen culturing, the ease of misdiagnosis, and the predominance of the disease in economically underdeveloped rural areas, leading to a potential severe underestimation of the actual number of infected cases. The clinical manifestations of human pythiosis vary, mainly occurring in corneal, vascular, and cutaneous types, usually invading the skin or mucosa, causing circular ulcerative granulomatous lesions, and leading to the inflammation of adjacent tissues. Histopathology is mainly manifested as eosinophilic granulomatous inflammation, and PAS staining and GMS staining can well display hyphae [4,5,6,7,8,9,10]. However, upon conducting a thorough literature review, to the best of our knowledge, there have been no reported cases of spinal pythiosis.

Here, we present a case of rare human spinal pythiosis diagnosed and treated in southern China. Although this is only a rare case study with certain limitations, we have found that there are currently no reports of *Pythium insidiosum* causing invasive infections in the deep organs of the human body both domestically and internationally. This case suggests that the pathogen can cause invasive infections of human deep organs, which will inevitably further exacerbate the harm of pythiosis. We are not yet sure on the following questions: Is it a mutant strain? Or does the body meet specific susceptibility conditions? A comprehensive review of this case and further research on the strain will undoubtedly help us better respond to the invasive infection of this pathogen, which is of great significance to human health.

Our patient might have contracted the infection by exposure to water contaminated with *Pythium insidiosum*, which does not rule out the possibility of an infection invasion caused by the pathogen penetrating the skin of the external auditory canal. Despite coexisting chronic hepatitis B and thalassemia, the patient’s immune function was not significantly impaired, with inflammatory markers such as PCT, CRP, and IL-6 remaining within normal ranges. Multiple tissue cultures yielded negative results, and pathological assessment indicated chronic suppurative inflammation only. Additionally, AF staining, GMS staining, and PAS staining were all negative for specific pathogens. These did not conform to the typical manifestations of most pythiosis. Notably, the patient’s condition fluctuated, marked by mild leukopenia, a markedly elevated ESR, and consistently high serum FER levels. Given the absence of definitive diagnostic indicators in routine tests and the failure of repeated microbiological examinations to confirm the diagnosis, we employed mNGS technology for pathogen screening. This approach successfully identified the rare *Pythium insidiosum*.

Timely and accurate diagnosis can significantly improve the prognosis of pythiosis. Given that *Pythium insidiosum* morphologically resembles filamentous fungi, traditional microbiological detection methods struggle to identify the organism. In terms of molecular biology analysis, there are mainly methods such as polymerase chain reaction (PCR), sequence homology, and proteomics, among which the most common method is PCR. However, it involves tedious processes such as primer design and sequencing steps, and PCR detection requires the prior knowledge or suspicion of a specific infection. As *Pythium insidiosum* is a rare pathogen, it is difficult to select appropriate PCR primers, and there is currently no definite, simple, and economical detection method. Therefore, for the detection of unknown rare pathogens, mNGS can detect almost all the known genomic sequences of pathogens in one test; although the cost is relatively expensive, the long-term benefits are greater [21,22,23,24,25,26,27,28,29,30,31,32]. From this case, it can also be seen that it was the timely intervention of mNGS that put the diagnosis and treatment process on track, reduced the risk of misdiagnosis and missed diagnosis, and may have had greater potential economic benefits.

Furthermore, to facilitate early and accurate pathogen identification, we initially compared various inoculation methods and cultivation conditions for clinical specimens. We found that only the inoculation methods using mycelial plugs and mycelial suspensions yielded satisfactory colony growth, while spore suspension, colony streaking, spreading, and puncturing inoculation methods failed to produce any visible growth. This suggests that for pythiosis infections inoculation and cultivation using tissue fragments or tissue homogenates are more conducive to obtaining positive results. We then proceeded to analyze the morphological characteristics of the strains, observing that the colonies were dominated by substrate mycelia, with hyphae extending into and expanding within the medium. This may explain why traditional colony streaking, spreading, and puncturing inoculation methods were unsuccessful in cultivating the organism. The growth curve indicated that the strain exhibited an expansive growth pattern on the LB agar after 48 h, with a notable decline in growth rate commencing on the 5th day post-inoculation, and by the 10th day, the colonies had covered a 90 mm plate, suggesting that the growth of *Pythium insidiosum* is somewhat slower than that of common pathogens. Clinical tissue specimens with a low microbial content may increase the possibility of missed detection only if they are cultured according to conventional procedures for 2–3 days. Consequently, the cultivation period for clinical tissue specimens can be extended from the conventional 2–3 days to 5–10 days, and if necessary, some media with high nutrient contents can be used to facilitate the growth of *Pythium insidiosum*.

In addition to colony morphology, lactophenol cotton blue staining and fluorescent staining were effective in identifying the *Pythium insidiosum*, clearly demonstrating its sparsely septate hyphae and sporangia. Notably, *Pythium insidiosum* exists in two forms: motile zoospore and hyphae, with the zoospore serving as the infectious propagules. Given the scarcity of reported methods for inducing sporulation and their limited efficacy, we innovatively utilized herbal juice to successfully induce sporulation, resulting in significantly increased sporulation rates and the easier isolation of the organism. Recognizing the genetic variability among *Pythium insidiosum* strains from diverse hosts and geographical locations, we intend to establish a comprehensive mass spectrometry database for local strains using matrix-assisted laser desorption/ionization time-of-flight mass spectrometry (MALDI-TOF MS). This cost-effective and straightforward method holds promise as the most economical and efficient means of identifying *Pythium insidiosum* [31,32].

Pythiosis, as a life-threatening condition, poses significant challenges for clinicians. The pursuit of not only early diagnostic methods but also effective antimicrobial therapies stand as a pivotal unresolved issue. Given the absence of ergosterol in the cell membrane of *Pythium insidiosum*, conventional antifungal agents are typically ineffective against this pathogen. Recent endeavors to treat pythiosis with drugs exhibiting antibacterial or antiparasitic activities, as well as bioactive components derived from medicinal plants and natural compounds, have yielded minimal to contradictory results [11,12,13,14,15]. In addition, *Pythium insidiosum* antigen (PIA) immunotherapy is an interesting therapy. To our knowledge, the process of *Pythium insidiosum* infection can lead to non-protective Th2-mediated immune responses. PIA can eliminate *Pythium insidiosum* by regulating the host immune response, but the induced antibody levels are influenced by various factors, including host immune status, underlying disease, the severity of infection, lesion size, and lesion location. In the face of the challenge posed by causative agents, hosts with a normal immune function may produce sufficient antibodies to eliminate the pathogen, while hosts with a certain degree of cellular immune dysfunction may need to be given granulocyte monocyte colony stimulating factors in addition to PIA vaccines to stimulate the immune response from Th2 to Th1, thereby enhancing the cytotoxic T cell killing of the pathogen. However, immunotherapy is still an auxiliary approach that requires the combination of other drugs or surgical treatment. Currently, the cornerstone of therapy relies heavily on extensive surgical excision, which is not only costly but may also result in lifelong disabilities and high recurrence rates [12,15,33,34].

The clinical management of *Pythium insidiosum* infections remains arduous, with no convincing therapeutic regimen. Drawing inspiration from the agar diffusion method documented in the literature [15,16,17], we devised a simplified paper disk-based antimicrobial susceptibility testing protocol to assess the in vitro sensitivity of strains to commonly prescribed medications. Our investigation revealed a modest sensitivity of this *Pythium insidiosum* strain to certain antibacterial agents, including oxazolidinones (linezolid), tetracyclines (tetracycline, doxycycline, tigecycline, and minocycline), macrolides (erythromycin and azithromycin), and phenicols (chloramphenicol). Further analysis using the broth microdilution method corroborated the lack of sensitivity to antifungal drugs, aligning with previous reports [11,12,15]. Among the aforementioned antibacterial agents exhibiting inhibitory effects, the MICs for therapeutic efficacy, such as linezolid (oxazolidinone), tetracycline and doxycycline (tetracyclines), erythromycin and azithromycin (macrolides), and chloramphenicol (phenicol), almost exceeded the upper limit of the safe concentration ranges for clinical treatment. In other words, the therapeutic concentrations required are prohibitively high, predisposing patients to adverse reactions. In the clinical course of this patient, we observed that, despite the initial administration of doxycycline and azithromycin at therapeutic doses, azithromycin was discontinued due to hearing loss and was replaced by TMP-SMX. Subsequently, the patient became intolerant to doxycycline and TMP-SMX, experiencing severe gastrointestinal reactions leading to self-discontinuation, with a subsequent disease exacerbation. Later, both doxycycline and TMP-SMX were withdrawn due to elevated creatinine levels, followed by the cessation of linezolid amidst markedly reduced blood cell counts.

After screening through drug sensitivity tests, we found that tigecycline, a tetracycline antibiotic and derivative of minocycline, exhibits excellent therapeutic efficacy with a moderate therapeutic concentration, making it a potential candidate for controlling *Pythium insidiosum* infection. Meanwhile, we attempted to conduct combined drug sensitivity tests based on tetracyclines. The results indicate that tetracyclines (including doxycycline, minocycline, and tigecycline) exhibit synergistic antimicrobial effects when combined with macrolides, oxazolidinones, or amphenicols. Although the specific mechanism of action of tetracyclines, macrolides, oxazolidinones, and amphenicols against *Pythium insidiosum* remains unclear, they share a similar primary mechanism against bacteria: mainly binding to different subunits of bacterial ribosomes, reducing the binding of amino acids and proteins, inhibiting protein synthesis, and inhibiting amino acid transport [35]. We observed a clear synergistic effect of these drug combinations on *Pythium insidiosum*, and it can be reasonably inferred that the binding site of the ribosome subunit of *Pythium insidiosum* may also be the target of the drug combination. However, as described earlier, combination therapy based on tetracyclines faces challenges such as a narrow therapeutic window and multiple adverse reactions, and there are also certain limitations in clinical application, which still require further research.

Interestingly, in vitro sensitivity tests showed that while TMP-SMX alone did not demonstrate effective antimicrobial activity, it exhibited antimicrobial effects when combined with doxycycline. In the previous treatments of patients with skin and subcutaneous pythiosis, TMP-SMX also played a crucial therapeutic role. We identified the *folP* gene encoding dihydropteroate synthase (DHPS), the receptor protein for sulfonamides, in the genome of *Pythium insidiosum* [5,36]. We speculate that TMP-SMX may inhibit the strain’s DHPS, preventing the pathogen from synthesizing the deoxyribonucleic acid and ribonucleic acid necessary for growth and reproduction.

Surprisingly, the MIC of the iron chelators deferiprone and deferoxamine against the strain was extremely low. During the course of the infection, which was never effectively controlled, the FER levels of the patient were extremely high, indicating a persistent state of iron overload. Iron overload is reported to enhance the invasive proliferation of microorganisms in host tissues. However, this effect is contingent upon the microorganisms’ ability to acquire and utilize iron [37]. It has been reported that the *FECH* gene, which encodes ferrochelatase, is presented in the genome of the *Pythium insidiosum* strain [38]. Based on this, it is reasonable to speculate that *Pythium insidiosum* enhances its ability to acquire utilizable iron by expressing ferrochelatase, thereby increasing the virulence of the pathogen. The intake of exogenous iron chelators can reduce excess iron in the body, competitively inhibiting the strain’s ferrochelatase from acquiring utilizable iron. This, in turn, can restore the body’s ability to control infections caused by *Pythium insidiosum*. While paying attention to the therapeutic potential of iron chelators, we should also recognize that the side effects and safety of iron chelators remain a focus of attention. They may cause dizziness, hypotension, and even serious side effects such as kidney, liver, and bone marrow failure. Long-term use may exacerbate adverse reactions and cause visual and hearing impairments. Therefore, regular checkups are required for blood cell levels, liver function, kidney function, as well as vision and hearing, emphasizing that the key to ensuring treatment safety is strictly monitoring and preventing the side effects of iron chelators [39].

It should be emphasized that “sensitive” drugs such as tetracyclines and macrolides observed in vitro drug sensitivity tests have not performed ideally in real-life cases, while “resistant” drugs like TMP-SMX have demonstrated good therapeutic effects [5]. This seemingly contradictory performance between in vitro and in vivo tests is due to several complex reasons, mainly including the following aspects. Firstly, the environments between in vitro and in vivo are different, and the influencing factors of in vitro experiments are single, only simply examining the effect of drugs on pathogens. Some pathogens can use some substances in the body to generate components that resist drugs, rendering drugs ineffective and leading to opposite sensitivity. Secondly, pathogens may alter their own structure in vivo, produce inactivating enzymes, form biofilms, and develop drug resistance. Thirdly, the pathological state and complex defense environment of the body may affect the absorption, distribution, metabolism, and excretion of drugs, affecting tissue concentration and ultimately affecting their efficacy. Lastly, current conventional drug susceptibility testing methods, such as disk diffusion susceptibility testing and broth microdilution, may not accurately reflect the drug sensitivity of *Pythium insidiosum* infection [40].

Nonetheless, the aforementioned discoveries provide us with new ideas for the treatment of pythiosis. We believe that combination therapy based on tetracyclines, especially tigecycline, the use of TMP-SMX, and the adjunctive use of iron chelators could all be potential treatment options for pythiosis. Given the chronic and recurrent nature of *Pythium insidiosum* infection, as well as the uniqueness of this case, further research, particularly in similar cases or in vivo experiments, is needed to validate solutions to this clinical challenge. It is also necessary to closely monitor the risks and side effects that may arise from excessive MIC or the expanded use of relevant drugs during the treatment process to ensure treatment safety.

## Figures and Tables

**Table 1:** Pathological testing during the course of illness ^a^.

Hospitalization Frequency	Anatomical Site	Microbiology	Pathology	mNGS (Sequences)
1st–4th Local Hospitals	blood/sputum/urine/stool/ left ear canal pus	(−)	NA	NA
5th Hospital I in Guangzhou	cervical lymph node and paravertebral abscess	(−)	slight infiltration of inflammatory cells, AF (−)	NA
6th Hospital II in Guangzhou	paravertebral cervical abscess	(−)	NA	48
	C3/4-C5/6 prevertebral/intervertebral/intraspinal lesions	(+)	chronic suppurative inflammation with abscess formation	1202
	C6/7-T2/3 intervertebral disk lesion	(−)	chronic active inflammation, AF (−), GMS (−), and PAS (−)	NA
7th Hospital II in Guangzhou	C7, T4/5-T7/8, and T11 intervertebral disk and paravertebral lesion	(+)	chronic suppurative inflammation, CD68 (+), Kappa (+), and Lambda (+)	0
8th Hospital II in Guangzhou	T9/10-T10/11 intervertebral disk and spinal canal infection lesion	(+)	vertebral abscess, AF (−), GMS (−), and PAS (−)	624

^a^ NA: data not available. (−): negative. (+): positive.

## Data Availability

The original fastq data underpinning the findings presented in this manuscript are accessible through the [NCBI] database under the accession number [PRJNA1123314]. The data can be accessed via the following link: https://www.ncbi.nlm.nih.gov/sra/PRJNA1123314 (accessed on 14 June 2024).
